# Normothermic and hypothermic oxygenated perfusion for donation after circulatory death in kidney transplantation: do we pay higher risk of severe infection after transplantation?: a case report

**DOI:** 10.1186/s12879-020-4835-0

**Published:** 2020-02-10

**Authors:** Matteo Ravaioli, Valeria Corradetti, Matteo Renzulli, Giuliana Germinario, Lorenzo Maroni, Federica Odaldi, Guido Fallani, Anna Paola Pezzuto, Daniele Parlanti, Raffaele Bova, Claudia Bini, Gaetano La Manna, Giorgia Comai

**Affiliations:** 1 Department of General Surgery and Transplantation, Sant’Orsola- Malpighi Hospital, University of Bologna, Via Massarenti 9, 40138 Bologna, Italy; 2 Department of Nephrology, S.Orsola- Malpighi Hospital, University of Bologna, Bologna, Italy; 3 Department of Radiology, S.Orsola- Malpighi Hospital, University of Bologna, Bologna, Italy

**Keywords:** Transplant, Infections, Arteritis, *Candida krusei*, *Pseudomonas aeruginosa*

## Abstract

**Background:**

Normothermic and hypothermic oxygenated perfusion for donation after circulatory death in kidney transplantation are becoming popular in Italy, with the purpose of reducing the risk of primary non function and delayed graft function due to the prolonged warm ischemia time.

Potential complications related to these procedures are currently under investigation and are continuously emerging with the increasing experience. Post-operative infections - in particular graft arteritis - are a rare complication but determine high risk of mortality and of graft loss.

The acute onset of the arterial complications makes it very difficult to find an effective treatment, and early diagnosis is crucial for saving both patient and graft. Prevention of such infections in this particular setting are advisable.

**Case presentation:**

We present a patient with an acute arterial rupture after transplantation of a DCD graft treated in-vivo hypothermic oxygenated perfusion. The cause was a severe arteritis of the renal artery caused by *Candida krusei* and *Pseudomonas aeruginosa*. We discussed our treatment and we compared it to the other reported series.

**Conclusion:**

Fungal infections in DCD transplant may be treacherous and strategies to prevent them should be advocated.

## Background

Post-transplant infections represent a major cause of morbidity and mortality. Pathogens may origin from either the donor or the recipient, and often show a predictable timeline of onset [1]. In the first post-transplant month gram-negative bacteria and fungi can determine a fatal outcome, especially in the case of invasive candidiasis, also because of its potentially insidious presentation [2].

Although data are not univocal, a higher infection rate has been reported in recipients of kidney transplants from donors after circulatory death (DCD) compared to donors after brain death (DBD) [3–5].

In this manuscript we report an acute arterial rupture of a DCD graft due to severe arteritis of the renal artery caused by *Candida krusei* and *Pseudomonas aeruginosa*.

## Case presentation

A 57-years-old patient received a DCD kidney after 2 years of peritoneal dialysis. The donor was a 60-years-old man, DCD Maastricht class III, died for cardiac arrest with an anamnesis of arterial hypertension. The donor was hospitalized for 7 days, 15 h and 19 min before explantation. Central venous catheter (CVC) blood cultures were positive for *E. coli* extended spectrum beta-lactamase (ESBL) producing, while blood cultures from peripheral vein were negative. Urine culture was negative and bronchoalveolar lavage (BAL) was positive for *E. coli*. No antibiotic therapy was performed. After three hours of extracorporeal membrane oxygenation (ECMO), the graft was treated ex-vivo with hypothermic oxygenated machine perfusion for 90 min, as of our center practice [6]. Pre-implantation biopsy reported a total Karpinsky score of 2 (interstitial fibrosis: 1, vascular disease: 1). The patient and the donor shared 3 human leukocyte antigens, one from class I and two from class II. A cefazolin-based perioperative therapy was started, and the graft was implanted in the right iliac fossa; upon reperfusion the graft showed immediately a good perfusion after eight hours of cold ischemia, and the peritoneal catheter was removed. Immunosuppressive therapy consisted in Basiliximab, steroids, tacrolimus, and mycophenolic acid. The immediate post-operative period was characterized by delayed graft function (DGF), requiring hemodialysis until post-transplant day 10. Four days after transplant, donor’s blood cultures resulted positive for a multi-susceptible *Escherichia coli*; moreover our patient developed a superficial infection of the surgical wound sustained by *Pseudomonas aeruginosa*, with persistently negative surveillance blood cultures.

The patient subsequently developed chill and fever with acute rise in C-reactive protein (CRP) and procalcitonin (PCT). Given the presence of sepsis, *P. aeruginosa* wound infection and *E. coli* ESBL-producing positive blood cultures from the donor, antibacterial therapy with meropenem – according to the donor’s antibiogram – was undertaken 8 days after kidney transplant. The initial dosage was 250 mg every 8 h and 1 g as extra-dose after each hemodialysis session.

**Fig. 1:**
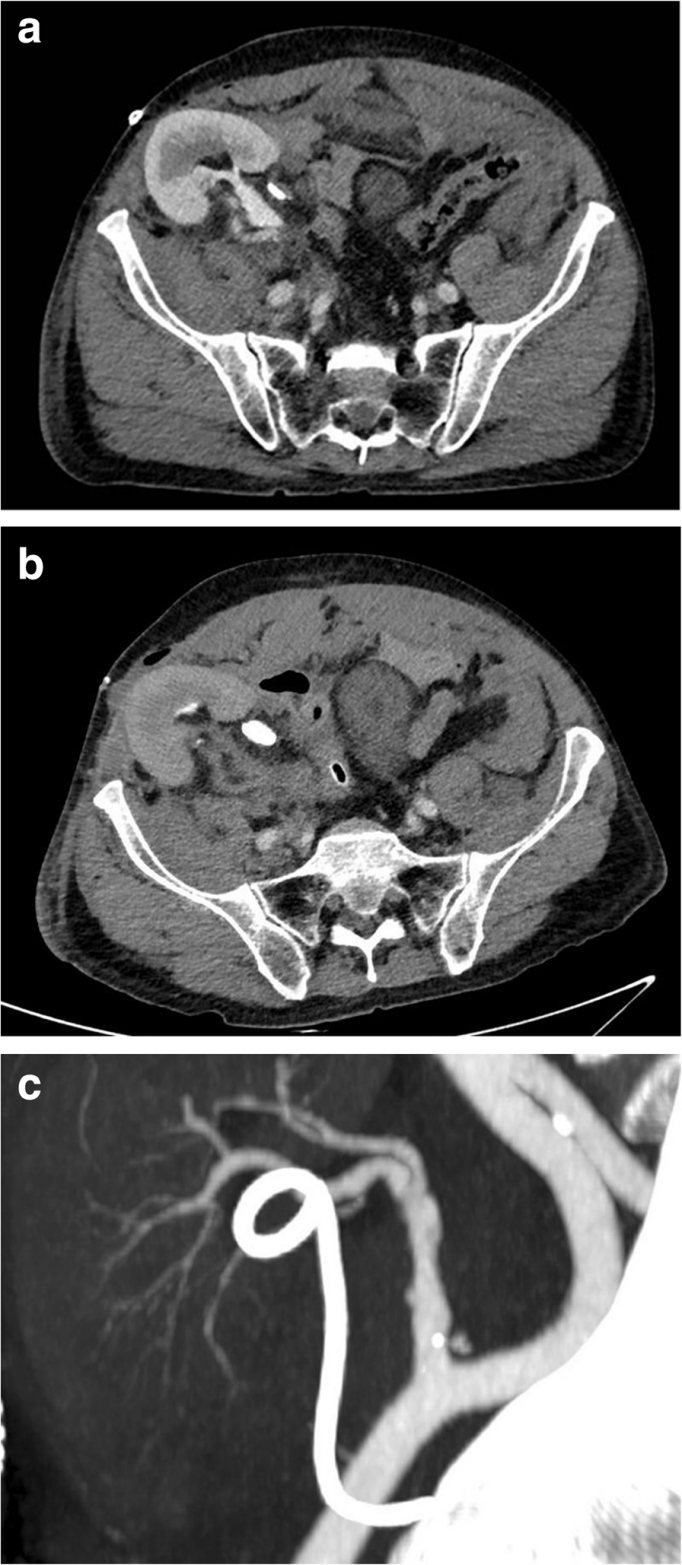
TC scan at different time: **a** at the time of patient pain, **b** few hours after the first CT scan and **c** revision of the imagining by an expert radiologist

The day before discharge the patient complained an acute abdominal pain; a computed tomography (CT) showed no remarkable findings, but a small collection in the right iliac fossa (Fig. 1a). White blood cells count and renal function were stable compared to the day before, and after a couple of hours the pain spontaneously improved; therefore no other procedures were planned. Seven hours later the pain acutely started again, and a second CT scan showed absence of blood flow in the artery of the graft, due to its complete rupture (Fig. 1b). Meropenem therapy was still ongoing in this phase. The patient was immediately brought to the operatory room, where a complete rupture of the renal artery was found between the anastomosis and the hilum, with massive hemorrhage. The graft was removed, and the iliac artery was subsequently clamped and repaired using a crio-preserved graft, with complete removal of the graft’s artery. Samples were collected and sent for histological and microbiological evaluation.

Upon suspicion of invasive fungal infection, anidulafungin therapy was started immediately after transplantectomy with a dosage of 100 mg every 24 h. Serum beta-D-glucan on blood samples collected after reintervention was 523.4 pg/ml.

Microbiological analyses on the artery wall resulted positive for *Pseudomonas aeruginosa* and *Candida krusei*, while cultures from the ureteral stent resulted positive for *Candida krusei*.

*Pseudomonas aeruginosa* isolated from the wound was similar to that isolated from the aneurysm: the antibiogram showed sensitivity to meropenem (MIC ≤0.25), piperacillin/tazobactam (MIC ≤2), gentamycin (MIC 2), ciprofloxacin (MIC 0.5), ceftazidime (MIC 2), cefepime (MIC 8) and amikacin (MIC 4).

The histology report concluded for a severe arteritis: an intense inflammatory reaction was present in both intima and avventitia, with a wide transmural necrotic area. The artery wall showed chronic and acute inflammation with focal necrosis and moderate arteriosclerosis with fibrosis and calcification, without documented hyphae.

Multiple antimicrobial therapy based on anidulafungin and meropenem was subsequently carried on for about 1 month. After reintervention the patient developed positive CVC blood cultures by *E. faecalis* and *S. epidermidis*, that did not require any further antimicrobial therapy implement.

Meropenem was continued until day 18 after transplantectomy with a fast improvement of cutaneous signs, white blood cells count, and serum CRP and PCT. Surveillance blood cultures resulted negative at the end of antibiotic therapy. Anidulafungin therapy was protracted for 2 months until negativization of serum beta-D-glucan.

Antimicrobial therapy management was discussed during multidisciplinary meetings which involved nephrologists, infectious diseases specialists and surgeons. The patient was strictly evaluated by the infectious disease team with almost daily evaluations.

After 6 months of follow up the patient is in good general conditions in extracorporeal hemodialysis; no other vascular or infective complications occurred and color-Doppler examination of the iliac vessels resulted normal.

Similar complications in the recipient of the other kidney were excluded by CT-scan and ultrasound (US), both negative for aneurysm or bleeding. The recipient of the other kidney did not develop positive blood cultures for Candida or *P. aeruginosa*; instead he developed positive blood cultures for *E. coli* and underwent antibiotic treatment with Ceftriaxone*.*

## Discussion and conclusion

Infections are common in kidney allograft recipients and according the time of onset show different epidemiology. In the first post-transplant period the severity of infections is related to both pathogen characteristics and the intense immunosuppression [1].

During the last years the necessity to expand the donor pool led to an increase of DCD utilization. This kind of transplants assure good outcomes in terms of graft function, except for a higher incidence of DGF and infective complications and lower patient survival [7, 8]. However, there is only partial evidence that post-transplant infection risk could be higher in DCD kidney transplants compared to the DBD ones [4, 8, 9]. In order to reduce complications related to longer warm and cold ischemia times, the DCD donor management is very complex and must be fast, due to the need of maintaining circulation during organ retrieval and of pre-implantation machine perfusion of organs [6, 8]. All of these steps represent possible contamination sources, that add to the already known potential infectious risk of the donor [10].

Infectious arteritis is a rare condition, presenting in patients immunocompromised or subject to invasive procedures. In the context of renal transplantation there are reports of graft artery aneurisms that presented at different post-transplant times, with variable clinical presentation, i.e. hypertension or declining renal function, and with the US evidence of pseudoaneurysm. In most cases the pathogen was a fungus already isolated from blood cultures of the recipient [11–19]. A study on a large number of donors, examining closely the possible source of infection, has demonstrated that patients who received a kidney with positive Candida *spp.* isolates from the donor, eventually developed candidiasis [19]. However, there are reported cases in which a known Candida *spp*. contamination in the donor did not exert any disease in the recipient [15].

In our patient the source of infection was difficult to define. In fact, we excluded contamination of the kidney perfusate since cultures resulted negative, and from donor’s blood cultures only a multi-sensitive *Escherichia coli* was isolated. Besides, in our patient the presence of an arterial pseudoaneurysm or aneurism was not detected by routine ultrasounds that we perform every other day. Moreover, the surveillance blood cultures we performed because of the donor *Escherichia coli* blood positivity resulted persistently negative.

Overall our patient presented with an unusual and insidious clinical picture as sometimes candidiasis does [2, 20]. In fact, we dealt with a severe disease that appeared suddenly without any of the typical signs of infection from Candida *spp.* or gram-negative E.coli ESBL or with local evidence of disease, i.e. US evidence of aneurism or collection.

Considering the potentially fatal outcome of arterial rupture due to infective arteritis with an atypical presentation, as in our case, this diagnostic hypothesis - in the setting of a donor and a graft that underwent invasive procedures - should be always considered. Of course, any attempt to identify a possible transmission from the donor must be achieved, even considering the potential low sensitivity and false negative results of cultures for fungi.

As a standard of care cultures of all organ preservation solutions and of donor’s blood and urine should be performed. Moreover, surveillance cultures of recipient’s blood, urine and drainage fluid of kidney surgical site should be carried out in presence of early symptoms of an active infection, in order to begin a targeted antimicrobial therapy as soon as possible.

Therefore anti-fungal prophylaxis should be taken in account in patients at high risk of invasive fungal infections, although it is difficult to determine which patients belong to this category [19].

Antifungal prophylaxis may be proposed to patients with risk factors, i.e., when preservation solution is positive for hyphae, when donor is colonized or when the recipient undergoes induction with thymoglobulin. Prophylaxis may be started just before transplantation on day 0 and antifungal prophylaxis using an echinocandin could be considered, although the data to support the use of a specific antifungal drug or the duration of the prophylaxis are currently scarce. Moreover, a prophylaxis of fungal infections, which have a low incidence rate, could expose the patient to resistance phenomena and potentially worsen the clinical picture [2, 10]. Certainly the best strategy is to stratify patients according to their risk.

On the other hand, the case of our patient shed light on the potential risk of fungal or E.coli ESBL infections using DCD managed with ECMO and ex-vivo perfusion [10, 21, 22]. In this setting an antifungal prophylaxis may be suggested, even if cultures from donor’s blood and from preservation fluids result negative.

Of course, a periodic US surveillance of graft anastomoses might be a valid strategy to prevent vascular complications, at least in cases where candidemia or sepsis hesitate in the formation of an arterial pseudoaneurysm or aneurism. In such cases, a strategy with either surgery or endovascular approach using covered stent can be planned and used as an effective method to prevent or stop bleeding, subsequently preserving the graft and patient survival.

In our case, even a prompt CT scan had not led to a definitive immediate diagnosis. In fact, only the revision of the first CT showed a minimal leakage in the arterial phase, that was probably due to a progressive rupture of the renal artery (Fig. 1c). At that time point a potential treatment with covered arterial stent or surgery with the replacement of the anastomosis could be considered [13, 14, 23].

The timing of diagnosis and surgical treatment is crucial to save the graft and the patient, but the surgical repair of the artery even with an autologous graft remains difficult to apply in an emergency setting.

Arteritis is a rare post-kidney transplant complication, but once occurred, is associated with high mortality and graft loss rate. The serious sequelae of these infections, mostly related to candidiasis, require an active post-transplant surveillance program in order to undertake a prompt targeted anti-infective prophylaxis or therapy. It is essential to repeatedly evaluate the patient with infectious disease specialists and to discuss the onset of each antimicrobial therapy after multidisciplinary meetings and discussions. In patients at high risk of infections, such as DCD recipients, an antifungal prophylaxis may be advocated. On the other hand, we need to consider that the application of ECMO and ex-situ organ perfusion in the setting of DCDs in Italy (which by law require 20 min of no touch period to declare circulatory death) is a novel clinical field with a low number of cases performed; for such reasons we do not know yet the actual rate of infection related to these procedure.

To date there is no evidence in literature, we can say that prophylaxis can be risky for everyone.

## Data Availability

No data or materials are available.

## Abbreviations

CT: Computed Tomography
DBD: Donors After Brain Death
DCD: Donors After Circulatory Death
DGF: Delayed Graft Function
ECMO: Extracorporeal Membrane Oxygenation
